# Human Resource and Funding Constraints for Essential Surgery in District Hospitals in Africa: A Retrospective Cross-Sectional Survey

**DOI:** 10.1371/journal.pmed.1000242

**Published:** 2010-03-09

**Authors:** Margaret E. Kruk, Andreas Wladis, Naboth Mbembati, S. Khady Ndao-Brumblay, Renee Y. Hsia, Moses Galukande, Sam Luboga, Alphonsus Matovu, Helder de Miranda, Doruk Ozgediz, Ana Romàn Quiñones, Peter C. Rockers, Johan von Schreeb, Fernando Vaz, Haile T. Debas, Sarah B. Macfarlane

**Affiliations:** 1 Department of Health Policy and Management, Mailman School of Public Health, Columbia University, New York, New York, United States of America; 2 Department of Surgery, Söder Hospital, Karolinska Institute, Stockholm, Sweden; 3 Department of Surgery, Muhimbili University of Health and Allied Sciences, Dar es Salaam, Tanzania; 4 Department of Health Management and Policy, University of Michigan School of Public Health, Ann Arbor, Michigan, United States of America; 5 Department of Emergency Medicine, University of California, San Francisco, California, United States of America; 6 Department of Surgery, College of Health Sciences, Makerere University, Kampala, Uganda; 7 Department of Anatomy, College of Health Sciences, Makerere University, Kampala, Uganda; 8 Department of Surgery, Kamuli Mission Hospital, Kamuli, Uganda; 9 School of Medicine, Catholic University of Mozambique, Beira, Mozambique; 10 Division of Pediatric Surgery, Hospital for Sick Children, University of Toronto, Toronto, Ontario, Canada; 11 Department of Epidemiology, School of Public Health, University of Michigan, Ann Arbor, Michigan, United States of America; 12 Division of International Health (IHCAR), Karolinska Institute, Stockholm, Sweden; 13 Higher Institute of Health Sciences, Maputo, Mozambique; 14 University of California, San Francisco, Global Health Sciences, San Francisco, California, United States of America; University of Queensland, Australia

## Abstract

**Background:**

There is a growing recognition that the provision of surgical services in low-income countries is inadequate to the need. While constrained health budgets and health worker shortages have been blamed for the low rates of surgery, there has been little empirical data on the providers of surgery and cost of surgical services in Africa. This study described the range of providers of surgical care and anesthesia and estimated the resources dedicated to surgery at district hospitals in three African countries.

**Methods and Findings:**

We conducted a retrospective cross-sectional survey of data from eight district hospitals in Mozambique, Tanzania, and Uganda. There were no specialist surgeons or anesthetists in any of the hospitals. Most of the health workers were nurses (77.5%), followed by mid-level providers (MLPs) not trained to provide surgical care (7.8%), and MLPs trained to perform surgical procedures (3.8%). There were one to six medical doctors per hospital (4.2% of clinical staff). Most major surgical procedures were performed by doctors (54.6%), however over one-third (35.9%) were done by MLPs. Anesthesia was mainly provided by nurses (39.4%). Most of the hospital expenditure was related to staffing. Of the total operating costs, only 7% to 14% was allocated to surgical care, the majority of which was for obstetric surgery. These costs represent a per capita expenditure on surgery ranging from US$0.05 to US$0.14 between the eight hospitals.

**Conclusion:**

African countries have adopted different policies to ensure the provision of surgical care in their respective district hospitals. Overall, the surgical output per capita was very low, reflecting low staffing ratios and limited expenditures for surgery. We found that most surgical and anesthesia services in the three countries in the study were provided by generalist doctors, MLPs, and nurses. Although more information is needed to estimate unmet need for surgery, increasing the funds allocated to surgery, and, in the absence of trained doctors and surgeons, formalizing the training of MLPs appears to be a pragmatic and cost-effective way to make basic surgical services available in underserved areas.

*Please see later in the article for the Editors' Summary*

## Abstract 2

In the second of two papers investigating surgical provision in eight district hospitals in Saharan African countries, Margaret Kruk and colleagues describe the range of providers of surgical care and anesthesia and estimate the related costs.

## Editors' Summary

**Background:**

Infectious diseases remain the major killers in developing countries, but traumatic injuries, complications of childbirth, and other conditions that need surgery are important contributors to the overall burden of disease in these countries. Unfortunately, the provision of surgical services in low- and middle-income countries is often insufficient. There are many fewer operations per a head of population in developing countries than in developed countries, essential operations such as cesarean sections for complicated deliveries are not always available, and elective operations such as male and female sterilization can be difficult to obtain. Lack of funding for surgical procedures and shortages of trained health workers have often been blamed for the low rates of surgery in developing countries. For example, anesthesiologists (doctors who are trained to give anesthetics and other pain-relieving agents) and trained anesthetists (usually nurses and technicians) are rare in many African countries, as are surgeons and obstetricians (doctors who look after women during pregnancy and childbirth). To make matters worse, these specialists often work in tertiary referral hospitals in large cities. In district hospitals, which provide most of the primary health care needs of rural populations, basic surgical care is usually provided by “mid-level health care providers” (MLPs)—individuals with a level of training between that of nurses and physicians.

**Why Was This Study Done?:**

Various organizations are currently working to improve emergency and essential surgical care in developing countries. For example, the Bellagio Essential Surgery Group (BESG) seeks to define, quantify, and address the problem of unmet surgical needs in sub-Saharan Africa. Importantly, however, before any programs can be introduced to improve access to surgical services in developing countries, better baseline data on existing surgical services needs to be collected—most of the available information on these services is anecdotal. In this study, the researchers (most of whom are members of the BESG) investigate the provision of surgical procedures and anesthesia in district hospitals in three sub-Saharan African countries and estimate the costs of surgery performed in the same hospitals.

**What Did the Researchers Do and Find?:**

The researchers collected recent data on the number of doctors, MLPs, and nurses in two district hospitals in Tanzania and in Mozambique, and from four district hospitals in Uganda and information on each hospital's expenditure. Most of the health workers in these hospitals (which care for 3 million people between them) were nurses (77.5%), followed by MLPs not trained to provide surgical care (7.8%), and MLPs trained to provide surgical care (3.8%). The hospitals had between one and six medical doctors each (28 across all the hospitals), but there were no trained surgeons or anesthesiologists posted at any of the hospitals. About half of the major surgical procedures undertaken at these hospitals were performed by doctors but more than a third were done by MLPs although the exact pattern of personnel involved in surgery varied among the three countries. Anesthesia was mostly provided by nurses and doctors; again the pattern of anesthesia provision varied among countries and hospitals. Only 7%–14% of overall hospital expenditure was allocated to surgical care and most of this allocation was used for obstetric services. Finally, the researchers estimate that, on the basis of district populations, the district hospitals spent between US$0.05 and US$0.14 per head on surgical services.

**What Do These Findings Mean?:**

These findings indicate that, in the district hospitals investigated in this study, physicians, MLPs, and nurses provide most of the surgical care. Furthermore, although all the hospitals in the study provide some surgical care, it accounts for a small part of the hospitals' overall operating costs. These findings may not be generalizable to other district hospitals in sub-Saharan Africa and provide no information about the unmet needs for surgical care. Nevertheless, these findings and those of a separate paper that investigates the range and volume of surgical procedures undertaken in the same district hospitals provide a valuable baseline for planning the expansion of health care services in Africa. They also suggest that increasing the funds allocated to surgery and formalizing the training of MLPs might be a cost-effective way of increasing access to surgical care in sub-Saharan Africa and other developing regions.

**Additional Information:**

Please access these Web sites via the online version of this summary at http://dx.doi.org/10.1371/journal.pmed.1000242.

- The range and volume of surgery in the same hospitals is investigated in a *PLoS Medicine* Research Article by Moses Galukande et al.
- Information on the Bellagio Essential Surgery Group is available
- WHO's Global initiative for Emergency and Essential Surgical Care plans to take essential emergency, basic surgery and anesthesia skills to health care staff in low- and middle-income countries around the world; WHO also has a page describing the importance of emergency and essential surgery in primary health care

## Introduction

There is a growing realization of the importance of surgical conditions in the overall burden of disease in low- and middle-income countries [1]–[5]. The Bellagio Essential Surgery Group (BESG), which met for the first time in June 2007 in Bellagio, Italy [6], seeks to define and quantify the problem of unmet surgical needs in sub-Saharan Africa (SSA) and the challenges to address it. Among other goals, BESG aims at producing evidence to address the large burden of surgically treatable diseases in SSA. The primary focus is to build capacity at district hospitals to increase availability of essential surgical and obstetrical services especially in emergency situations.

The evidence for the health system response to surgical conditions and injuries in the developing world is primarily anecdotal; however, published statistics on surgical service provision suggest that service provision is not adequate to address need [7],[8]. Care for surgical conditions and injuries is particularly scarce in SSA where shortages of skilled health care providers, including surgeons, anesthetists, and nurses; low levels of health funding; and poor physical infrastructure have limited the ability of health systems to reliably provide even the most basic surgical services [9]–[11]. For example, in high-income countries the estimated mean rate of surgeries performed is 11,110 per 100,000 population annually, while the corresponding figure in low-income countries is 295 [12].

A severe shortage of skilled health care providers contributes to a significant deficit in surgical output in many low-income countries, particularly in SSA [13]. District hospitals should be capable of providing basic surgical procedures [8], but lack of resources and of qualified staff are main deterrents for those in need, unless they can afford to self-refer to tertiary hospitals. A prospective study of the referral system in Dar es Salaam, Tanzania, for example, found that over 70% of over 11,000 patients seen at the National Referral Hospital were self-referrals having bypassed the district hospital and 67% of these were for surgical problems [14]. In this study, lack of expertise at district hospitals was cited in 96% of the cases as the reason for referral to a higher level-hospital.

There are few specialist surgeons and anesthetists in SSA and they usually work in specialized tertiary referral hospitals in large cities. For instance, Uganda has only ten specialist surgeons and 350 anesthetists to serve a population of over 30 million people [15]. Although generalist physicians are trained to perform basic surgery and induce anesthesia in some SSA countries, even they are scarce at district hospitals. Consequently, most health care in these district hospitals is provided by a cadre of mid-level health care practitioners (MLPs), with a level of training between that of a nurse and a physician. MLPs are often trained to subspecialise and in some countries they are trained to undertake basic surgical procedures. A recent review found MLPs working in 25 of 47 SSA countries [16]. These MLPs performed minor surgery in 12 of these 25 countries, but they only performed major surgery (including cesarean sections and orthopedic procedures) in seven, and anesthesia in four of these countries (Table 1). In Malawi, clinical officers currently perform the vast majority of major surgical procedures in district and central hospitals [11],[17]. Another longstanding example is Mozambique, which started training “técnicos de cirurgia” in basic surgical procedures, and anesthesia in 1984 [18]. These surgically trained nurses and medical assistants are now widely accepted in the Mozambican health system and perform over 90% of all obstetric surgeries in Mozambique, a country with around 800 physicians (including expatriates) and 30 obstetricians to serve a population of about 21 million people [19],[20].

**Table 1:** MLPs doing surgery/anesthesia in SSA.

Countries	Surgical Services Provided	Anesthesia	Preservice Training (y)
**Angola**	Minor surgery	—	3
**Burkina Faso**	Minor surgery	—	3
**Ethiopia**	Minor surgery, cesarean section	—	3
**Ghana**	Minor surgery, cesarean section	—	3
**Kenya**	Minor surgery, orthopedics	Yes	3
**Malawi**	Minor surgery, cesarean section, orthopedics	—	3
**Mozambique**	Minor surgery, cesarean section, abdominal surgery	Yes	3
**Senegal**	Minor surgery	—	N/A
**Sudan**	Minor surgery	—	3
**Tanzania**	Minor surgery, cesarean section, abdominal surgery, orthopedics	—	2
**Togo**	Minor surgery	—	2
**Uganda**	Minor surgery	Yes	3
**Zambia**	Orthopedics	Yes	3

[16]. N/A, not applicable.

Limited health care funding for surgical care is also a major constraint to providing surgical care in SSA [7],[21]. Surgical care is systematically omitted from essential health care packages in Africa. Although this omission is likely fuelled by the perception that it is prohibitively expensive, the existing evidence suggests that basic surgery in a district hospital context can be cost-effective and can compare favorably with other well-accepted health interventions, including vaccination and care for HIV/AIDS [21]–[23]. Cost-effectiveness of basic surgical procedures can be as little as US$70 to US$230 per disability-adjusted life year (DALY) averted [24], with interventions ranging from acute care, head or burn injuries, to elective surgery for easily corrected conditions such as cataracts that can exact a large toll on individuals and their families [5],[25]. It is, however, difficult for national health policymakers and their international partners to assess the cost of incorporating surgery into routine service delivery without an understanding of the costs for delivering surgical services in the current African context. This assessment is especially important as more dollars are being spent on health systems in low-income countries with the focus on strengthening health systems [26]–[28].

This is the second of two articles describing the surgical care provided in eight district hospitals in Mozambique, Tanzania, and Uganda [29]. In the first paper, we observed that, whereas surgical output varied between hospitals, all hospitals provided major surgery such as cesarean sections and herniorrhaphies. This article expands on these findings by (1) characterizing the range of health care providers of surgical procedures and anesthesia; and (2) estimating attributable costs of surgery performed in the same district hospitals. This information will provide baseline data of existing services for future planning and for a global dialogue on human and financial investments needed to strengthen surgical service provision in SSA.

## Methods

### Selection Criteria

We selected hospitals in three countries represented at the inaugural meeting of the BESG: Tanzania, Uganda, and Mozambique. The choice of three countries allowed us to assess variability in inputs, service provision, and costs of services in different settings. To permit comparability across countries, hospitals selected for the study were required to be designated district hospitals both funded and operated by the government. We chose rural hospitals that had 50–150 beds, had been in operation for at least 2 y, and were not located in the capital city (where referral patterns could be expected to differ substantially). Finally, these hospitals had to have cost and utilization data available for 2007 and/or 2006. The hospitals selected ranged from rural and remote, such as Kasulu in Tanzania and Catandica in Mozambique, to hospitals proximate to the capital city: Bagamoyo in Tanzania, Chokwe in Mozambique, and Mityana and Kiryandongo in Uganda. More information about the contextual setting for the selected countries and hospitals can be found in the first article of the series [29].

### Data Collection

Data were collected from hospital records, hospital administrator interviews, and district administrative records. District demographics such as district population were gathered from the most recent census in each country. The number of other hospitals and health centers in the district was obtained from local hospital administrators.

At the hospital level, we collected comprehensive information about human resources in each hospital: number of doctors, surgeons, dentists, technicians or assistant medical officers, clinical officers or agents, nurses (including the subcategories of nurse officers, nurses' assistants, enrolled nurses, nurse midwives, public health nurses), and administrative and support personnel. These data were taken from hospital personnel rolls and confirmed by hospital administrators. Data on other hospital inputs (e.g., functioning and nonfunctioning beds, operating rooms, etc.) also came from central administration records in each hospital.

Data on admissions and procedures were collected from two sources. First, we collected aggregate hospital statistics on annual admissions, deliveries, and major surgeries from existing hospital information systems (which produce monthly statistics). These data were collected for 2007, and 2006 when 2007 data were not available. Second, for the target months of February, June, and October 2007, we reviewed ward-level patient records from medical, surgical, maternity, and pediatric wards to determine admission diagnosis, age, and gender as well operating room logs for surgical procedure details. We also recorded the type of procedure (if any), and the category of health worker who performed it. To standardize data entry and case definition, all sites used the same list of most common procedures and diagnoses on the basis of a consensus panel of physicians in the respective countries. A category “other” was only possible if none of the listed choices were appropriate, and in those cases, an open-ended description was required. Third, we collected estimates for typical lengths-of-stay for the major medical and surgical diagnoses from two local physicians and surgeons at each site to determine total inpatient days for each category of admissions.

Annual recurrent expenditures were collected from each hospital and converted to 2007 US$ using the International Monetary Fund Gross Domestic Product (IMF GDP) deflator [30]. These expenditures were collected according to categories of salaries (and overtime) for each level of personnel (including employment allowances such as travel, moving expenses, and uniforms), medical and dental benefits, training, goods, medical supplies, laboratory supplies, fuels and lubricants, grounds maintenance/cleaning, equipment maintenance, office materials (nondurable and durable), services, communications, funeral subsidies, insurance, utilities, water, electricity, telephone/internet, waste disposal, and other. These expenses were obtained from annual planned expenditure reports in some cases, and monthly expenditures in the remaining cases. The latter were adjusted to an annual timeframe. We further collected information about annual direct donations to the hospitals from hospital administrators.

Ethical clearance was not required as all data were de-identified administrative data.

### Data Analysis

Two separate analyses using Excel and SPSS [31],[32] were performed to describe the providers of surgical care, and to estimate health care and surgical care expenditures.

#### Providers of surgical care

For these analyses, patient-level information on surgical procedures performed for the targeted months of February, June, and October were used. Additionally, information on the health professionals who performed the surgery and provided anesthesia, if applicable, was included in the analysis. The extent to which these selected months are representative of the procedures performed throughout the year is discussed in the first paper of the series [29]. Surgical procedures were grouped into major (obstetric and nonobstetric) surgeries, and minor surgeries. More details about this classification are also available in the first paper of the series [29].

Four sets of descriptive statistics are presented: the titles, roles, and responsibilites of MLPs in SSA, and the countries studied; hospital staffing in each site; distribution of surgical procedures by type of providers for each site; and anesthesia by type of provider.

#### Cost of surgical care

To estimate cost of surgical care, basic descriptive statistics were calculated, including service ratios and hospital spending per district capita. The analyses were performed in two steps. First the overall cost of inpatient and outpatient services provided was estimated. In the second step, the cost of surgical and nonsurgical inpatient services provided was estimated. We deliberately used individual-level data (3-mo) to estimate cost of surgical care instead of aggregate-level for the purpose of applying the average length of stay specific to each inpatient diagnosis.

In the first step, because hospitals did not track expenditures by inpatient versus outpatient services, we estimated the allocation of hospital expenditures to outpatient and inpatient services under different assumptions, on the basis of reports from hospital administrators. Administrators estimated that between 20% and 30% of non-personnel–operating costs of the hospital were expended on outpatient clinics (e.g., diagnostic tests, utilities, maintenance of equipment, medicines). Given an average of three outpatient clinics per week, lasting 6–8 h each, they further estimated that between 30% and 50% of personnel time was devoted to outpatient care. We therefore calculated the total cost of inpatient care under these different assumptions. The more conservative model allocated 50% of personnel cost and 70% of non-personnel cost to inpatient services; the more liberal model allocated 70% of personnel cost and 80% of non-personnel cost to inpatient services. Most findings presented in the results section rely on the conservative model.

In the second step, we estimated the allocation of hospital expenditures to inpatient surgical and nonsurgical services. We used average lengths of stay for each medical and surgical inpatient diagnosis to calculate total inpatient days attributable to surgical and medical conditions at each hospital. The proportion of inpatient days attributable to each diagnosis was then used to apportion the total expenditures for each study site. It is important to note that this method, although informative, is likely to underestimate the higher intensity of capital resources required to treat surgical versus medical patients (e.g., surgical supplies, utilities, anesthetics). Minor surgeries including circumcisions, incision and drainage, surgical toilet and suture, wound debridement and dressing, and Norplant insertion and removal were included in our cost analysis under the outpatient care rubric. Cost estimates were not produced for Buluba and Iganga because of missing information.

## Results

### Providers of Surgical Care

Eight rural district hospitals were included in the study: Chokwe and Catandica in Mozambique, Kasulu and Bagamoyo in Tanzania, and Mityana, Kiryandongo, Buluba, and Iganga in Uganda. The number of people served by the hospitals ranged from 137,582 to 626,742 (Table 2). The aggregated population of these districts amounts to approximately 2,900,000 people.

**Table 2:** Distribution of health care facilities by study site and population served.

Site and Population	Tanzania		Mozambique		Uganda^a^			
**Country population^b^**	40,200,000		21,300,000		31,400,000			
**District in the study**	Bagamoyo	Kasulu	Barue	Chokwe	Mityana	Masindi	Mayuge	Iganga
**District hospital in the study**	Bagamoyo	Kasulu	Catandica	Chokwe	Mityana	Kiryandongo	Buluba	Iganga
**District population**	228,814^c^	626,742^c^	137,582^d^	187,422^d^	269,763^c^	459,490^c^	326,567	547,155
**Number of district hospitals**	1	1	1	1	1	1	1	1
**Other hospitals in the district**	0	2	0	0	0	0	0	0

a Ugandan hospitals are referred to as “General Hospitals” rather than “District Hospitals.” The “Other” hospital in Mubende district is another “General” hospital.

b [41].

c 2002 country-level census data.

d 2007 country-level census data.

Table 3 shows the training and scope of clinical activities of MLPs working in the three countries, with substantial heterogeneity across countries. Table 4 provides a compilation of human resources in each hospital. Altogether 28 doctors were available to the above population, which amounts to about 0.01 physicians/1,000 people. Between one and six medical doctors were available per hospital but there were no trained surgeons posted at any of the district hospitals. However, one of the Ugandan hospitals (Buluba) was served sporadically by a specialist surgeon from the nearest regional hospital and the Bagamoyo Hospital had periodic eye surgery camps attended by visiting ophthalmological surgeons. Overall 4.8% of the medical staff were doctors (and two dentists), 82.8% were nurses, and 12.4% were MLPs.

**Table 3:** Training and clinical scope of practice of MLPs in Tanzania, Mozambique, and Uganda.

Country and MLP	Training	Scope of Practice
**Tanzania**		
Assistant medical officers	5–6 y postsecondary education	Provide surgical, obstetric, medical, pediatric, and community health services at the district hospital.
Clinical officers	3 y postsecondary education	Small surgical procedures.
		Assist Medical Officer at DH In charge of health centres.
Anesthetic officers	5–6 y postsecondary education (must also take AMO general course)	Provide anesthetic services at district/regional hospital.
**Mozambique**		
*Técnicos de cirurgia*	Program started in 1984	90% of all obstetric surgeries in Mozambique.
	Selected among nurses and medical assistants	Capacity to handle surgery, complications of deliveries, gynecology, and trauma conditions.
	2 y postsecondary theoretical and practical instruction	
	1-y internship under the supervision of a surgeon in a district hospital	
Clinical officers	2.5 y postsecondary training	Medicine, minor surgery, obstetrics, anesthesia.
**Uganda**		
Clinical officers	3 y postsecondary training	Medicine, and pediatric outpatient care, hospice care. May assist with medical and pediatric care in absence of doctor.
		In charge of some health centers.
		Diagnose surgical, obstetric, and gynecological conditions and refer them to the doctor.
Anesthetist officers	3 y postsecondary training	Administer anesthesia to adults, infants, and children. Deployed in the national and regional referral hospitals. Are the main anesthetists in these hospitals.
Orthopedic officers	3 y postsecondary training	Basic orthopedic procedures and management of club foot (the Ponsetti manouevre).

**Table 4:** Human resources distribution in study hospitals.

Hospital Positions	Bagamoyo (TZ)	Kasulu (TZ)	Catandicaa,b (MZ)	Chokwe^b^ (MZ)	Mityana (UG)	Kiryandongo (UG)	Buluba (UG)	Iganga (UG)
**Licensed medical practitioners**								
Doctors (general practitioners)	5	1	1	4	6	2	4	5
Surgeons	0	0	0	0	0	0	0	0
Dentists	1	0	0	1	0	0	0	0
**MLPs**								
Assistant medical officers^b^	8	13	3	1	0	0	0	0
Clinical officers	11	11	4	3	9	3	3	8
Nurses (total)	(88)	(116)	(18)	(31)	(73)	(75)	(47)	(68)
Nurse officers	20	18	—	—	32	9	9	19
Nurse assistants	37^c^	56	5	—	12	26	21	18
Enrolled nurses	0	5	11	—	10	18	9	17
Nurse midwives	22	35	2	—	19	22	8	14
Public health nurses	9	2	0	0	0	0	—	—
Administrators/support	3	6	2	3	8	4	10	4
**Total**	116	147	28	43	96	84	67	85

a The classification of nurses in Catandica may not fully correspond to the study classification; of the 18 nurses two were general nurses with mid-level training, two were maternity and child health nurses with mid-level training, three were nurses with elementary level training, and 11 were nurses with basic level nurses. Nurses with mid-level training were classified as nurse assistants, nurses with elementary training and nurses with basic level skills were classified as enrolled nurses, and maternal and child health nurses were classified as nurse midwives.

b In Mozambique, assistant medical officers performing surgery are referred to as “técnicos de cirurgia.”

c Nurse assistants in Bagamoyo are referred to as medical attendants.

MZ, Mozambique; TZ, Tanzania; UG, Uganda.

**Figure 1:**
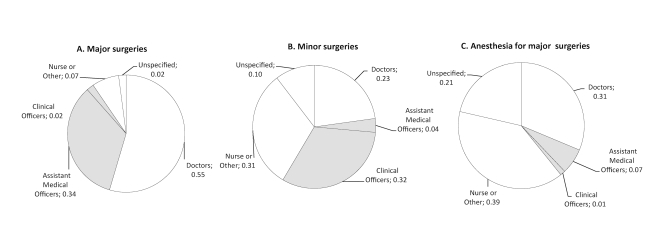
Providers of major surgery, minor surgery, and anesthesia across eight hospitals in three Sub-Saharan African countries. (A) Major surgeries; (B) minor surgeries; (C) anesthesia for major surgeries. Note: MLPs highlighted.

Overall, MLPs contributed 35.9% of both major and minor surgeries, but only 7.9% of anesthesia (Figure 1A–1C). Nurses provided anesthesia in nearly 40% of the surgical procedures. Table 5 shows the breakdown of major and minor surgical procedures by site and provider type. A total of 1,706 surgical procedures were recorded in the district hospitals studied during the study period, 79.4% of which were major surgeries, including obstetrics surgeries. These data give a crude annual surgical rate of 235/100,000 people.

**Table 5:** Surgical procedures performed by type of medical provider for three selected months (February, June, and October).

Procedure and Provider	Bagamoyo (TZ)^a^	Kasulu (TZ)^a^	Catandica (MZ)a,b	Chokwe (MZ)a,b	Kiryandongo (UG)^c^	Mityana (UG)^c^	Buluba (UG)^c^	Iganga (UG)^c^	Total (%)
**Total surgeries performed in the three selected months**									
Major surgeries	262	198	50	99	44	265	83	353	1354 (79.4)
Minor surgeries	4	—	3	1	33	104	6	42	193 (11.3)
Unspecified surgeries^d^	—	—	—	—	3	—	—	156	159 (9.3)
Total	266	198	53	100	80	369	89	551	1706 (100.0)
**Major surgeries (including OB) by type of provider**									
Doctor (general practitioners)	2 (0.8)	29 (14.6)	—	40 (40.4)	18 (40.9)	248 (93.6)	52 (74.7)	340 (96.3)	739 (54.6)
Assistant medical officers	199 (76.0)	159 (80.3)	50 (100.0)	51 (51.5)	—	—	—	—	459 (33.9)
Clinical officer	1 (0.4)	—	—	—	14 (31.8)	12 (4.5)	—	—	27 (2.0)
Nurse or other	60 (22.9)	10 (5.1)	—	8 (8.1)	2 (5.4)	2 (0.8)	18 (21.7)	1 (0.3)	101 (7.5)
Unspecified	—	—	—	—	10 (21.6)	3 (1.1)	3 (3.6)	12 (3.4)	28 (2.1)
**Minor surgeries by type of provider**									
Doctor (general practitioners)	—	—	—	—	1 (2.5)	3 (2.9)	5 (83.3)	35 (83.3)	44 (22.8)
Assistant medical officers	3 (75.0)	—	3 (100.0)	1 (100.0)	—	—	—	—	7 (3.6)
Clinical officer	—	—	—	—	20 (62.5)	42 (40.4)	—	—	62 (32.1)
Nurse or other	1 (25.0)	—	—	—	4 (10.0)	51 (49.0)	1 (16.7)	3 (7.1)	60 (31.1)
Unspecified	—	—	—	—	8 (25.0)	8 (7.7)	—	4 (9.5)	20 (10.4)

a Surgical procedures are for February, June, and October 2007.

b Surgical procedures are underrepresented in October for Mozambique, where medical staff undergoes training during that month: there were no procedures for Catandica compared to a monthly average of 20 procedures, and 16 in Chokwe compared to 50 monthly average.

c Surgical procedures are for February, June, and October 2006.

d 137 unspecified surgeries were performed by doctors, three by assistant medical officers, 16 by nurses/others, and three providers were not identified.

MZ, Mozambique; OB, obstetric; TZ,Tanzania; UG, Uganda.

While most of the major procedures were performed by medical doctors (54.6%), a substantial number was attributable to MLPs (35.9%) and nurses (7.5%). There were considerable differences between the three countries, and whereas most of the major surgical procedures were carried out by medical doctors in Uganda (range, 40.9%–96.3%), such procedures were for the most part done by MLPs in Mozambique and Tanzania (range 51.5% to 100%). Even within countries, there were conspicuous differences. Accordingly, 100% of major procedures were performed by MLPs in Catandica Hospital in Mozambique, whereas the corresponding figure for the other Mozambican hospital was 51.5%. Conversely, in two of the four Ugandan hospitals, major surgery was performed by doctors in over 90% of the cases (range, 40.9%–96.3%). In Tanzania, it is notable that in the hospital with five employed doctors (Bagamoyo), 76% of major surgeries were carried out by MLPs, while in the other hospital, which had only one doctor the corresponding level was 80%. Minor surgeries were evenly distributed among MLPs (35.7%), nurses (31.1%), and medical doctors (22.8%) (Table 5).

Data on the provision of anesthesia are provided in Table 6, which indicates within and across country variations. Overall, most anesthesia was provided by nurses (39.4%) or doctors (31.3%). In Tanzania, nursing assistants provided anesthesia in 100% of procedures at Bagamoyo, and 79.8% of major procedures at Kasulu with an assistant medical officer (AMO) anesthetist providing the rest (20.2%). In Uganda, data on the provision of anesthesia was not complete in all hospitals except Mityana and Kiryandongo, but anesthesia was provided by a mixture of doctors, clinical officers, and nurses. In Mozambique anesthesia was given solely by assistant medical officers in Catandica, and by nurses in Chokwe.

**Table 6:** Anesthesia provided by type of medical provider for three selected months (February, June, and October).

Anesthesia and Provider	Bagamoyo (TZ)^a^	Kasulu (TZ)^a^	Catandica^b^ (MZ)	Chokwe^b^ (MZ)	Kiryandongo (UG)^c^	Mityana (UG)^c^	Buluba (UG)^c^	Iganga (UG)^c^	Total (%)
**Anesthesia for major surgeries (including OB) by type of provider**									
Doctors (general practitioners)	—	—	—	—	3 (6.8)	71 (26.8)	—	350 (99.2)	424 (31.3)
Assistant medical officers	—	40 (20.2)	50 (100.0)	—	—	—	—	—	107 (7.9)
Clinical officer	—	—	—	—	17 (38.6)	—	—	—	—
Nurse or other	262 (100.0)	158 (79.8)	—	98 (99.0)	14 (31.8)	1 (0.4)	—	—	533 (39.4)
NA/unspecified	—	—	—	1 (1.0)	10 (22.7)	193 (72.8)	83 (100.0)	3 (0.8)	290 (21.4)
**Anesthesia for minor surgeries by type of provider**									
Doctors (general practitioners)	—	—	—	—	1 (3.0)	—	—	42 (100.0)	43 (22.3)
Assistant medical officers	—	—	3 (100.0)	—	—	—	—	—	26 (13.5)
Clinical officer	—	—	—	—	18 (54.5)	5 (4.8)	—	—	—
Nurse or other	4 (100.0)	—	—	1 (100.0)	6 (18.2)	11 (10.6)	—	—	22 (11.4)
NA/unspecified	—	—	—	—	8 (24.2)	88 (84.6)	6 (100.0)	—	102 (52.8)

a Surgical procedures are for February, June, and October 2007.

b Surgical procedures are under-represented in October for Mozambique, where medical staff undergoes training during that month: there were no procedures for Catandica compared to a monthly average of 20 procedures, and 16 in Chokwe compared to 50 monthly average.

c Surgical procedures are for February, June, and October 2006.

MZ, Mozambique; OB, obstetric; TZ, Tanzania; UG, Uganda.

### Cost of Surgical Care

The overall annual operating expenditures range from US$155,908 in Catandica, Mozambique to US$800,662 in Kasulu, Tanzania. Inpatient operating expenditures were between 53% and 63% of the annual operating expenditures under the conservative assumption (72%–77% under the liberal assumption). In most hospitals, personnel was the largest cost category for inpatient care, comprising on average 60.3% of operating costs across hospitals (ranging from 35% to 83%). Purchases funded or given directly to the hospital by donors are not included in these estimates as these can vary widely from year to year. Many of these donations are cash or in-kind donations for capital equipment. For example, Catandica hospital received an operating table, operating theater lights, and some surgical equipment worth approximately US$30,000 in 2007. Kasulu Hospital received approximately US$190,000 of donated equipment and medicines from local and international nongovernmental organizations (NGOs). Although the focus in this study was on regular, budgeted spending, it is important to note that additional donor funds, when used for operating expenses, can substantially increase hospitals' financial resources.

Table 7 shows the per admission and per inpatient day operating costs of the hospital. It also shows how operating expenditures were allocated to medical and surgical inpatient services. We found under the conservative assumption, that between 7% (US$11,376) and 14% (US$33,980) of operating costs were attributable to surgeries. Obstetric surgery, particularly cesarean section, was a substantial component of surgical costs in all hospitals (between 3% and 8%). Lastly, we calculated the hospital surgical spending per capita, on the basis of district populations, and found that overall spending on hospital services ranged from US$0.05 to US$0.14 per capita. The estimate remains between US$0.06 and US$0.19 under the least conservative assumption.

**Table 7:** District hospital average recurrent expenditures by volume and type of inpatient service.

Expenditures	Bagamoyo (TZ)		Kasulu (TZ)		Chokwe (MZ)		Catandica (MZ)		Mityana (UG)		Kiryandongo (UG)	
	US$	Percent	US$	Percent	US$	Percent	US$	Percent	US$	Percent	US$	Percent
**Overall hospital expenditures** a												
Per capita (district)	1.44	—	1.28	—	1.53	—	1.13	—	0.36	—	0.80	—
Per admission	53.02	—	85.24	—	43.33	—	42.11	—	39.96	—	68.81	—
Per inpatient day	17.70	—	26.84	—	10.79	—	18.16	—	11.22	—	22.05	—
**Overall surgical expenditures** b												
Per surgery	56.41	—	98.82	—	41.54	—	49.03	—	55.34	—	304.28	—
Per capita	0.14	—	0.13	—	0.10	—	0.08	—	0.05	—	0.07	—
**Hospital expenditures by category of inpatient service** c,d												
Total surgical services	31,700	10	84,492	11	19,358	7	11,376	7	33,980	14	33,470	9
Obstetric surgery	16,255	5	25,445	3	7,201	3	5,472	4	16,033	6	31,109	8
General surgery	15,445	5	59,047	7	12,157	4	5,904	4	17,947	7	2,361	1
Total nonsurgical services	144,662	44	376,951	47	146,611	51	82,575	53	124,452	49	181,498	49
Normal deliveries	25,505	8	64,875	8	30,078	10	40,219	26	39,928	16	28,927	8
Medical diagnoses	119,157	36	312,076	39	116,533	41	42,356	27	84,524	34	152,571	41
**Total operating expenditures**	329,716	—	800,662	—	286,593	—	155,908	—	251,448	—	369,419	—

a The numerator is overall annual hospital expenditures.

b The numerator is overall inpatient surgical expenditures.

c For each percentage, the numerator is the expenditures for each inpatient service, and the denominator is the overall annual operating expenditures.

d Cost allocation is done under the conservative assumption that 50% of personnel cost and 70% of non-personnel costs are attributable to inpatient care.

MZ, Mozambique; TZ,Tanzania; UG, Uganda.

## Discussion

The current study describes the providers of and expenditures for surgical care in three sub-Saharan African countries. We confirm that nurses, MLPs, and generalist physicians constitute the back-bone of surgical care provision in Tanzania, Uganda, and Mozambique. We further found that while some surgical care is provided in all these district hospitals, it only accounts for a small portion of hospital operating costs in the hospitals under study. Surgical care for approximately 3 million people living in the districts served by the hospitals in this study was provided by generalist medical doctors and MLPs. No specialist surgeon or anesthesiologist was available on a regular basis. Notably, nearly half of the major surgeries were carried out by nondoctors. Nurses were essential as well, providing anesthesia in four of ten major surgical procedures. We also found a very low surgical rate per 100,000 people and per medical staff performing surgery.

Although the countries in the study are comparable in terms of size, per capita income, and basic public health indicators, they have chosen to address the need for qualified surgical providers differently. Mozambique relies heavily on *técnicos de cirurgia* and Tanzania on assistant medical officers, whereas Uganda relies to a greater degree on doctors (generalists). It is unknown to what extent these approaches reflect and adequately address the need for surgery. A recent study estimated that the surgical rates per 100,000 people for the three countries in the current study was 338 for Tanzania, 267 for Mozambique, and 161 for Uganda [12]. These differences in surgical rates may suggest that MLPs have an important role to play in increasing access to essential surgery in SSA. Studies in Mozambique and Malawi have shown comparable health outcomes when comparing surgeries performed by MLPs with gynecologists and medical officers [17],[33]. Training *técnicos de cirurgia* has proven cost effective when compared with educating surgeons and gynecologists [34]. The retention of *técnicos de cirurgia* in the districts is also much higher [20]. The strategy of using MLPs to provide essential surgical care in district hospitals appears to be the only solution until these countries are able to train adequate numbers of doctors and specialists.

Anesthesia is a prerequisite for surgery. The lack of anesthetists and anesthetic technicians in SSA compounds the difficulties in delivering safe surgery in district hospitals with much of avoidable perioperative mortality stemming from anesthesia-related complications [6],[35]–[37]. In our study, anesthesia in major surgery was induced most commonly by nurses or generalist doctors. As with the provision of surgery, there were differences between the countries in the study. While there was no doctor to induce anesthesia in Mozambique or Tanzania, this was the case in every third major procedure in Uganda. It is unknown how these different approaches to provide anesthesia affect access to and quality of services given.

As expected, we found that the majority of hospital expenditures were allocated to care for medical (versus surgical or obstetric) conditions in the hospitals in our study. Although this may be partly a function of lower demand for surgery compared to medical treatment, it is also likely a reflection of the relatively limited availability of surgical and anesthesia personnel as described above; but also of other core inputs, such as reliable drug supplies, electricity, etc.

Across the three sub-Saharan African countries, hospital operating cost for surgery was between US$11,376 and US$84,492, representing 7% to 14% of the overall hospital expenditures. Thus, despite small hospital budgets, a great deal of surgery is already being done at low costs. The cost per major surgical procedure ranged between US$40 in a hospital with average surgical volume and US$300 in a hospital with low surgical volume. This cost is relatively modest compared to the per episode cost for delivery of complex interventions currently implemented at large scale in Africa, which involves both diagnosis and long-term treatment. For example, the cost of one hospital visit for a patient infected with HIV was US$41.70 in South Africa and in south-east Nigeria. Patients on antiretroviral treatment spent an additional US$95.10 on diagnostic/laboratory costs per month [38],[39]. It is important to emphasize that these costs are for chronic conditions and represent ongoing expenses, whereas most surgical interventions are curative, and as such, performed only once.

The relatively low cost per surgery could also be related to the use of mid-level health workers. However, given that personnel constitutes half or more of operating costs and concurrently, volume of surgical output, we expect that appropriate surgical staffing, with an emphasis on medically trained surgeons and anesthetists, may substantially raise the costs of surgical care delivery.

It was recently projected that it would take 36 y or more for SSA countries to reach the WHO target for physician density [40]. No study has estimated the time required to staff hospitals across SSA with sufficient numbers of qualified surgeons and anesthetists to meet minimum requirements, as such minimum requirements have not been defined. Population-based data on the burden of surgical disease burden in SSA are necessary to estimate the need for essential surgery. Such estimates are necessary for appropriate health policy adaptations. The surgical DALYs lost per 1,000 population are reported to be highest in the world in Africa, but this estimate was founded on a “near total lack of pertinent data” [8].

Until enough trained surgeons and anesthetists are available to serve the populations of SSA, trained MLPs and nurses will likely be required to increase access to essential surgery. As is apparent from this study, MLPs already play a pivotal role in surgical care at the district hospital. Formalizing their training, clearly defining the delegation of clinical responsibility, ensuring supervision, and attention to accreditation and licensing will all be required to ensure high quality care. For example, ensuring consistent adoption of practice guidelines on the use of regional (e.g., spinal) in place of general anesthesia for many surgical procedures should permit greater participation of trained MLPs and reduce operative morbidity. National and regional surgical societies and other stakeholders such as academic institutions and NGOs have to be involved in the process of recognizing MLPs and provide support for the development of core technical and clinical competencies for MLPs. On the basis of experiences from different SSA countries, the programs should be geared towards health staff with basic medical education, have a targeted professional scope, and be achieved within 3–4 y. To do this, acceptance and support by medical and surgical associations in the respective countries is critical. The worst outcome is to create a parallel system of ill-trained, ill-supervised MLP surgeons with little interaction with trained surgeons. Such a system would result in a system of inferior surgical care in SSA.

Findings from this study should be interpreted in the light of several limitations. First, the study only documents three SSA countries and eight district hospitals, which may not represent the realities of other African countries. The results may also underestimate the total provision of surgical care, given that two small mission hospitals in one of the eight districts were not included in the analysis. Procedure-related data were only collected for 3 mo and from two different years. Second, this study is potentially limited by the accuracy of available utilization and expenditure data in each facility. The operating room log book entries were not always uniform across sites, and there are always possibilities of inaccurate reporting related to issues of omission, transmission, commission, and transcription. In particular, minor procedures were likely to be under-recorded given that the majority of these took place outside the operating room. Specific to the expenditures data, we attempted to corroborate annual spending figures with data from previous years to ensure the data chosen for analysis were representative. However, as noted above, the figures presented here do not include cash and in-kind donations made directly to the hospitals. Where direct donations consist of (or are spent on) capital purchases, these would not affect operating expenditure estimates, although off-budget drug purchases, for example, would mean our operating expenditure estimates are biased downward.

The cost estimates do not include out-of pocket expenses, nor do they include indirect costs. For instance, it is customary for patients to purchase medications and use laboratory and diagnostic services outside the hospital premises. Also, indirect costs such as family involvement in patient care were not included in the analysis. Thus, our cost estimates are conservative, but there is no reason to believe that the bias is unevenly distributed between inpatient and outpatient services. Finally, the estimates could be further underestimated for surgical services given the assumption that all patient days incur the same costs across medical and surgical admissions.

### Conclusion

Our data suggest that greater financial and human resource investment in strengthening the capacity of district hospitals to deliver essential surgery is a potentially neglected opportunity to achieve important gains in population health in low-income settings. We found a critical shortage of physicians across the the eight hospitals, with only 28 physicians serving a population of 3 million. Our findings further highlight the instrumental role played by trained MLPs and nurses in the provision of surgical care in SSA. In addition to minor surgeries, both major surgeries and anesthesia are provided by a combination of medical officers, MLPs, and nurses. It will be essential to clearly define the scope of practice, supervision processes, and quality standards for all cadres of providers, to ensure that expanding the number and range of surgical and anesthesia providers will result in concomitant improvements in access to and quality of essential surgical care in SSA.

## Abbreviations

MLP: mid-level provider
SSA: sub-Saharan Africa
